# Remnant renal volume can predict prognosis of remnant renal function in kidney transplantation donors: a prospective observational study

**DOI:** 10.1186/s12882-021-02568-8

**Published:** 2021-11-06

**Authors:** Shunta Hori, Nobumichi Tanaka, Tatsuo Yoneda, Nobutaka Nishimura, Mitsuru Tomizawa, Tomonori Nakahama, Yasushi Nakai, Makito Miyake, Kazumasa Torimoto, Kiyoyuki Minamiguchi, Kiyohide Fujimoto

**Affiliations:** 1 Department of Urology, Nara Medical University, 840 Shijo-cho, Kashihara, Nara, 634-8522 Japan; 2 Department of Radiology and Nuclear Medicine, Nara Medical University, 840 Shijo-cho, Kashihara, Nara, 634-8522 Japan

**Keywords:** Computed tomography volumetry, Donor selection, Living donation, Living donor kidney transplantation, Remnant renal function, Remnant renal volume, Renal scintigraphy

## Abstract

**Background:**

Safety and survival during and after donor nephrectomy (DN) are one of the main concerns in living kidney donors (LKDs). Therefore, kidney (left/right) to be procured should be determined after considering the difficulty of DN, as well as the preservation of remnant renal function (RRF). In this prospective study, we investigated the roles of computed tomography volumetry (CTV) in split renal function (SRF) and established a predictive model for RRF in LKDs.

**Methods:**

We assessed 103 LKDs who underwent DN at our institute. The Volume Analyzer SYNAPSE VINCENT image analysis system were used as CTV. RRF was defined as the estimated glomerular filtration rate (eGFR) 12 months after DN. The association between various factors measured by CTV and RRF were investigated, and a role of CTV on prediction for RRF was assessed.

**Results:**

The median age and the preoperative eGFR were 58 years and 80.7 mL/min/1.73m^2^, respectively. Each factor measured by CTV showed an association with RRF. The ratio of remnant renal volume to body surface area (RRV/BSA) could predict RRF. In addition, RRV/BSA could predict RRF more accurately when used together with age and 24-h creatinine clearance (CrCl).

**Conclusions:**

Our findings suggest that RRV/BSA measured by CTV can play an important role in predicting RRF, and a comprehensive assessment including age and CrCl is important to determine the kidney to be procured.

**Supplementary Information:**

The online version contains supplementary material available at 10.1186/s12882-021-02568-8.

## Background

Living donor kidney transplantation (LDKT) provides a better graft and patient outcome compared to deceased donor kidney transplantation (KT) [1]. The highest priority in LDKT is the safety and health of LKDs during and after donor nephrectomy (DN). Therefore, the careful consideration should be given to the decision to donate, i.e. the procured kidney side. In this regard, split renal function (SRF) and anatomical suitability are important factors to be considered in the selection of donated kidney (DK). In particular, the degree of difficulty during surgery is associated with the outcome immediately after KT in both recipients and LKDs. In addition, remnant renal function (RRF) is closely correlated with the risk of end-stage renal disease (ESRD), cardiovascular disease, and cerebrovascular disease after DN, which consequently affect the prognosis of LKDs [2–5].

Recently, computed tomography (CT) or magnetic resonance imaging volumetry, known as functional renal volume, has been reported as a useful assessment tool for SRF that can predict RRF in LKDs after DN, and in patients with renal cell carcinoma after nephrectomy [6–15]. Sanusi et al. showed that renal volume was correlated with renal function and could be a predictive factor for the development of chronic kidney disease (CKD) [16]. In Japan, SRF is frequently evaluated using nuclear renography and/or CT volumetry (CTV). In our institute, 99 m-Tc diethylenetriamine penta-acetic acid (DTPA) scintigraphy has been used for the evaluation of SRF before DN; 99 m-Tc DTPA scintigraphy can measure the split glomerular filtration rate (GFR). However, several limitations of nuclear renography have been reported, such as long procedure duration, exposure to radioisotopes, and imaging artifacts, leading to inaccurate evaluation of SRF. Therefore, CTV has been investigated as an alternative reliable surrogate marker for SRF [6–12, 17].

The present study aimed to evaluate 99 m-Tc DTPA scintigraphy and CTV for their ability to accurately predict RRF in LKDs after DN in order to suggest a predictive model for RRF. This is not intended to predict actual value of RRF but to predict a population with well-maintained RRF after DN. An understanding of the roles and advantages of CTV in obtaining information, including not only anatomical evaluation but also SRF, can lead to improved selection of DK, and decrease in the risk to LKDs during and after DN.

## Methods

### Patient selection, data collection, and study design

**Fig. 1:**
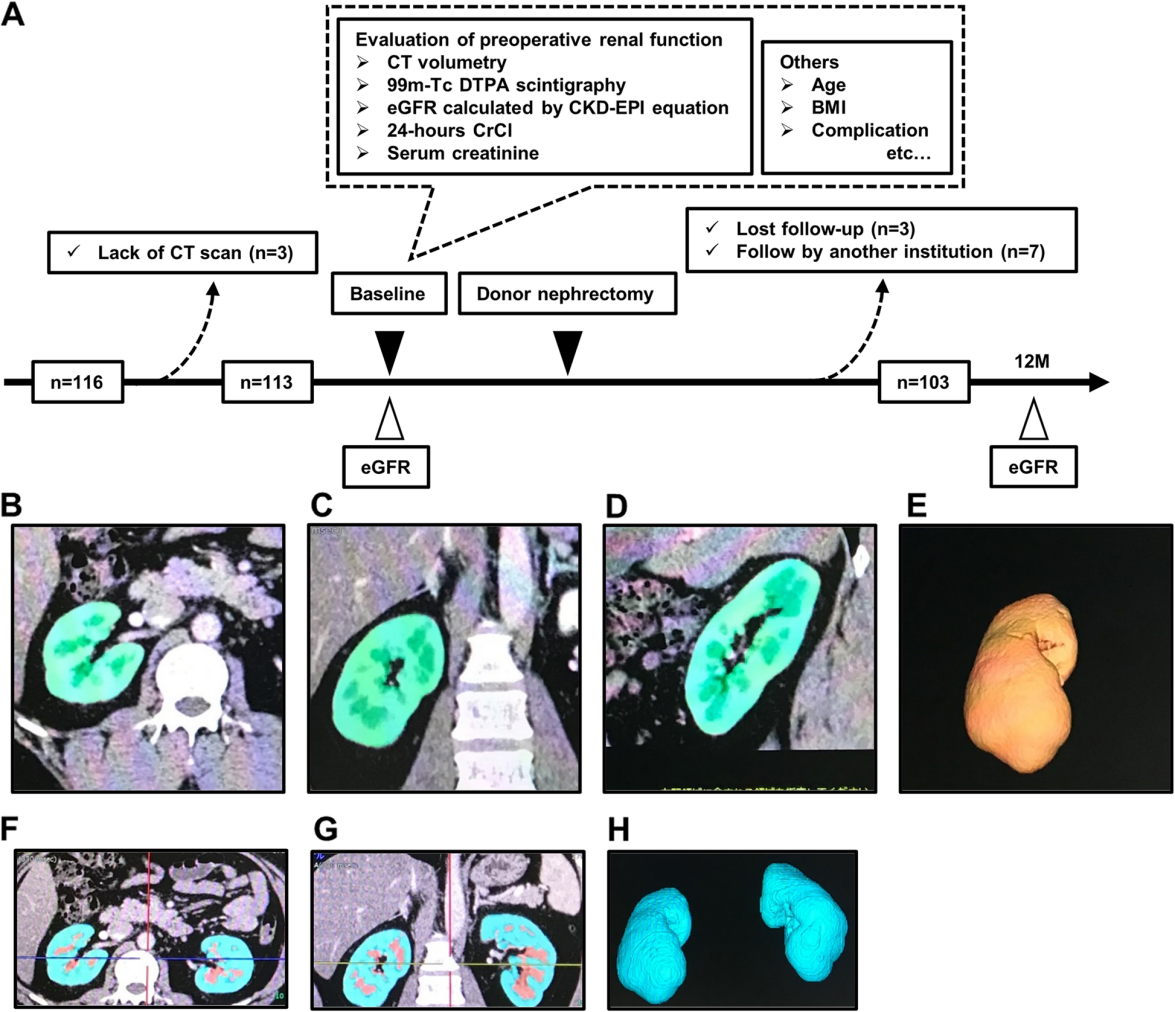
Study workflow and representative images obtained from the Volume Analyzer SYNAPSE VINCENT image analysis system. A total of 116 consecutive living kidney donors (LKDs) were enrolled, and 13 LKDs were excluded due to a lack of data and/or a short follow-up period. The medical information of the remaining 103 LKDs was obtained prospectively. The estimated glomerular filtration rate (eGFR), 24-h creatinine clearance, serum creatinine, renal volume calculated by computed tomography volumetry, and renal scintigraphy were used for preoperative assessment of renal function. The eGFR 12 months after donor nephrectomy was defined as remnant renal function (**A**). Representative images obtained from the Volume Analyzer SYNAPSE VINCENT image analysis system are shown. The renal parenchymal volume (**B**; axial image, **C**; coronal, **D**; sagittal image, **E**; 3-dimmentional image) and cortex volume (**F**; axial image, **G**; coronal image, **H**; 3-dimmentinal image) were measured automatically. CT: Computed tomography, DTPA: Diethylenetriamine penta-acetic acid, eGFR: Estimated glomerular filtration rate, CKD-EPI: Chronic Kidney Disease Epidemiology Collaboration, CrCl: Creatinine clearance, BMI: Body mass index, M: Months

The study protocol was approved by the Institutional Review Board for Clinical Studies of Nara Medical University (Medical Ethics Committee ID: NMU-323-4), and written informed consent was obtained from all LKDs following an explanation of the study objectives and protocol. Figure 1A shows a flow diagram of the present study. The objectives of this prospective study were LKDs, and enhanced CT and 99 m-Tc DTPA scintigraphy were performed preoperatively. The estimated GFR (eGFR), calculated using the Chronic Kidney Disease Epidemiology Collaboration equation [18], was evaluated 12 months after DN. The eGFR was used as a measure of RRF for analysis, and 99 m-Tc DTPA scintigraphy and CTV were performed to determine the more accurate method of the two in predicting RRF in the short-term after DN.

### Measurement of renal volume and renal cortex volume

Enhanced CT images obtained for preoperative screening and examination of vascular structures were analyzed using the Volume Analyzer SYNAPSE VINCENT image analysis system (Fujifilm Medical, Tokyo, Japan) to quantify renal volume and renal cortex volume. All LKDs were evaluated with a 64-slice multi-detector CT scanner (SOMATOM® Definition AS, Siemens Medical Solutions, Erlangen, Germany) and triple-phase CT images (plain, arterial phase, nephrographic phase) were obtained. During the scan, the contrast agent was injected at a rate of 3 mL/s, and arterial and nephrographic phases were performed 30 and 120 s, respectively, after the start of contrast injection. Figure 1B, C, and D show the renal parenchymal area in the axial, coronal, and sagittal reconstruction, respectively. Figure 1E shows a representative three-dimensional image of the renal parenchyma. Figure 1F and G show the renal cortex area in the axial and coronal reconstructions using the arterial phase, respectively. Figure 1H shows a representative three-dimensional image of the renal cortex. Both renal parenchymal and cortical volumes were analyzed automatically and immediately. Abnormal structures, including space-occupying lesions, such as renal cysts, infarction, aneurysm, or mass were automatically excluded from the analysis. The measured values were adjusted by body surface area (BSA) or body weight (BW); BSA was calculated according to the DuBois-DuBois formula.

### Protocol for 99 m-Tc diethylenetriamine penta-acetic acid scintigraphy

In our institute, 99 m-Tc DTPA scintigraphy was used for the assessment of SRF. The LKDs were examined in the supine position with their back against the gamma camera (SYMBIA, Canon Inc., Tokyo, Japan), and 99 m-Tc DTPA was injected intravenously. Scintigraphy can measure bilateral GFR (measured GFR: mGFR) due to the excretion of DTPA from the glomerulus, which results in the determination of the laterality of the DN. The kidney is procured from the inferior side when SRF of the kidneys differ by more than 10%.

### Definition of remnant renal function

The primary outcome of this study was RRF at 1 year after DN. We defined RRF to be well preserved if the eGFR at 1 year after DN was > 60% of the preoperative eGFR and poorly preserved if the eGFR at 1 year after DN was ≤60% of the preoperative eGFR. The cut-off value was determined according to previous studies, which suggested that an eGFR in the range of 62.5–67% of the preoperative renal function was considered to indicate good RRF [19].

### Statistical analysis

Statistical analyses were performed and figures were plotted using GraphPad Prism 7.0 (GraphPad Software, San Diego, CA, USA). The interrelationship between each factor was examined using Spearman’s rank correlation coefficient. Receiver operating characteristic (ROC) curve analysis was performed to identify predictive factors for RRF after DN, and the area under the curve was assessed. The optimal cut-off value for maximizing the sum of the sensitivity and specificity was determined as the point closest to the upper left-hand corner. EZR (Easy R) statistical software was used to compare the ROC curves [20]. Two-sided tests were used in all cases, and a *P* value < 0.05 was considered to indicate statistical significance in all analyses.

## Results

### Patient characteristics

A total of 116 consecutive LKDs who underwent DN between April 2008 and December 2018 at our institute were enrolled. Among these, 13 LKDs (11.2%) were excluded from the analysis due to a lack of preoperative CT images and/or a follow-up period of < 12 months. Finally, we evaluated the medical information of 103 LKDs, and obtained clinical and radiographic data. Table 1 shows the baseline clinical characteristics, preoperative renal function, and comorbidities for the cohort comprising 103 LKDs. The median age and BMI at DN were 58 years and 23.0 kg/m,^2^ respectively (interquartile range [IQR], 22–75 and 18.2–34.9, respectively). A BMI ≥ 30 kg/m^2^ was identified in 5 LKDs, 4 of whom were men. The preoperative median serum creatinine level and eGFR were 0.65 mg/dL and 80.7 mL/min/1.73m^2^, respectively (IQR, 0.45–1.08, and 58.7–128.2, respectively). This cohort of LKDs comprised 39 men (38%) and 64 women (62%). No LKD showed proteinuria ≥150 mg/gCr, and a Charlson comorbidity index ≥1 was observed in 4 LKDs (4%). Hypertension, diabetes, hyperlipidemia, and hyperuricemia were observed in 13 (13%), 4 (4%), 11 (11%), and 4 (4%) LKDs, respectively. Around 80% of DN procedures were performed via hand-assisted laparoscopic donor nephrectomy. No patient experienced blood loss requiring transfusion, and no major perioperative complications (> grade 2 of the Clavien classification (Table S1) [21]) were reported in any patient. Approximately 80% of LKDs were procured from the left kidney as the DK. New-onset hypertension, diabetes, or proteinuria were not observed in any patient during a 1-year follow-up, and no progression was observed in 4 LKDs with known diabetes.

**Table 1:** Patients’ background

Variables		Number of donors	%
**Total**		**103**	**100**
**Age at operation**	**Median (IQR)**	**58 (22–75)**	**–**
**Height (m)**	**Median (IQR)**	**1.60 (1.55–1.68)**	**–**
**Body Weight (kg)**	**Median (IQR)**	**60.0 (51.0–67.4)**	**–**
**BMI (kg/m**^**2**^**)**	**Median (IQR)**	**23.0 (18.2–34.9)**	**–**
**BSA (m**^**2**^**)**	**Median (IQR)**	**1.64 (1.50–1.73)**	**–**
**Serum creatinine (mg/dL)**	**Median (IQR)**	**0.65 (0.45–1.08)**	**–**
**eGFR (mL/min/1.73m**^**2**^**)**	**Median (IQR)**	**80.7 (58.7–128.2)**	**–**
**24-h CrCl**	**Median (IQR)**	**99.4 (86.2–118.1)**	**–**
**Sex**	**Male**	**39**	**38**
	**Female**	**64**	**62**
**CCI**	**0**	**99**	**96**
	**≥1**	**4**	**4**
**Hypertension**	**No**	**90**	**87**
	**Yes**	**13**	**13**
**Diabetes**	**No**	**99**	**96**
	**Yes**	**4**	**4**
**Hyperlipidemia**	**No**	**92**	**89**
	**Yes**	**11**	**11**
**Hyperuricemia**	**No**	**99**	**96**
	**Yes**	**4**	**4**
**Type of donor nephrectomy**	**HALDN**	**83**	**81**
	**Open**	**20**	**19**
**Procured side**	**Left**	**86**	**83**
	**Right**	**17**	**17**

*IQR* interquartile range, *BMI* body mass index, *BSA* body surface area, *eGFR* estimate glomerular filtration rate, *CrCl* creatinine clearance, *CCI* Charlson comorbidity index, *HALDN* hand-assist laparoscopic donor nephrectomy

### Baseline values of split renal parenchymal, cortex volumes, and measured glomerular filtration rate

The preoperative baseline median (IQR) and mean. ± standard deviation values for each volume and mGFR are shown in Table 2. The median donated renal volume (DRV), remnant renal volume (RRV), donated renal cortex volume (DRCV), and remnant renal cortex volume (RRCV) were 147.3 mL, 143.4 mL, 102.5 mL, and 101.9 mL, respectively (IQR, 131.2–168.8, 129.9–167.6, 88.4–118.0, and 89.0–120.9, respectively). There was no significant difference between the DRV and RRV parameters (*P* = 0.71 and *P* = 0.78, respectively). There was also no significant difference in volume parameters adjusted by BW or BSA between the DRV and RRV parameters (DRV/BW vs. RRV/BW; *P* = 0.66, DRCV/BW vs. RRCV/BW; *P* = 0.83, DRV/BSA vs. RRV/BSA; *P* = 0.37, and DRCV/BSA vs RRCV/BSA; *P* = 0.99, respectively). In addition, the median mGFR of the DK and remnant kidney (RK) was 46.8 and 45.1, respectively (IQR, 37.7–52.8, and 38.8–52.3, respectively; *P* = 0.91). Overall, there was no laterality of SRF, as measured by 99 m-Tc DTPA scintigraphy and CTV, between DK and RK.

**Table 2:** Baseline split renal volumes and measured glomerular filtration rate

Variables	Median (IQR)	Mean ± SD	***P*** value
**DRV (mL)**	**147.3 (131.2–168.8)**	**149.9 ± 30.5**	**0.71**
**RRV (mL)**	**143.4 (129.9–167.6)**	**148.9 ± 31.2**	
**DRCV (mL)**	**102.5 (88.4–118.0)**	**105.4 ± 27.4**	**0.78**
**RRCV (mL)**	**101.9 (89.0–120.9)**	**107.0 ± 27.0**	
**DRV/BW (mL/kg)**	**2.46 (2.21–2.71)**	**2.47 ± 0.41**	**0.66**
**RRV/BW (mL/kg)**	**2.41 (2.19–2.67)**	**2.46 ± 0.43**	
**DRCV/BW (mL/kg)**	**1.71 (1.48–1.93)**	**1.73 ± 0.35**	**0.83**
**RRCV/BW (mL/kg)**	**1.75 (1.47–1.91)**	**1.69 ± 0.45**	
**DRV/BSA (mL/m**^**2**^**)**	**90.5 (82.5–98.7)**	**91.1 ± 13.8**	**0.37**
**RRV/BSA (mL/m**^**2**^**)**	**88.9 (81.6–96.9)**	**89.8 ± 13.0**	
**DRCV/BSA (mL/m**^**2**^**)**	**63.2 (54.4–72.6)**	**63.8 ± 13.0**	**0.99**
**RRCV/BSA (mL/m**^**2**^**)**	**63.0 (55.1–70.4)**	**62.2 ± 16.1**	
**mGFR of DK**	**46.8 (37.7–52.8)**	**45.9 ± 10.9**	**0.91**
**mGFR of RK**	**45.1 (38.8–52.3)**	**46.3 ± 10.6**	

*IQR* interquartile range, *SD* standard deviation, *DRV* donated renal volume, *RRV* remnant renal volume, *DRCV* donated renal cortex volume, *RRCV* remnant renal cortex volume, *mGFR* measured glomerular filtration rate, *DK* donated kidney, *RK* remnant kidney

### Correlation analyses between preoperative factors and remnant renal function

Correlation analyses were performed to evaluate the effect of preoperative factors on RRF (Table 3). Age had a negative correlation with RRF (Spearman ρ = − 0.49, *P* <  0.0001), while sex showed no correlation (Spearman ρ = 0.058, *P* = 0.56). Preoperative factors involved in renal function, such as serum creatinine, eGFR, and 24-h creatinine clearance (CrCl) were significantly correlated with RRF (Spearman ρ = − 0.48, *P* <  0.0001; Spearman ρ = 0.60, *P* <  0.0001; Spearman ρ = 0.34, *P* = 0.0004, respectively). Preoperative factors calculated by CTV were significantly correlated with RRF regardless of DK or RK, and RRV/BW and RRV/BSA had a stronger correlation than the other markers (RRV/BW; Spearman r = 0.54, *P* <  0.0001, and RRV/BSA; Spearman ρ = 0.48, *P* <  0.0001). Furthermore, the mGFR of the DK and RK was also significantly correlated with RRF (Spearman ρ = 0.29, *P* = 0.0036 and Spearman ρ = 0.41, *P* <  0.0001, respectively). In contrast, the measured weight of the DK during surgery was not correlated with RRF (Spearman ρ = 0.015, *P* = 0.88).

**Table 3:** Correlation between eGFR 1 year after donor nephrectomy and preoperative variables

Variables	Spearman ρ	***P*** value
**Age**	**−0.49**	**< 0.0001**
**Sex**	**0.058**	**0.56**
**Serum creatinine**	**−0.48**	**< 0.0001**
**eGFR**	**0.6**	**< 0.0001**
**24-h CrCl**	**0.34**	**0.0004**
**Height**	**0.051**	**0.61**
**BW**	**−0.14**	**0.17**
**BMI**	**−0.23**	**0.022**
**BSA**	**−0.074**	**0.46**
**DRV**	**0.26**	**0.0073**
**DRCV**	**0.29**	**0.0029**
**RRV**	**0.33**	**0.0006**
**RRCV**	**0.35**	**0.0004**
**RRV/BW**	**0.54**	**< 0.0001**
**RRCV/BW**	**0.47**	**< 0.0001**
**RRV/BSA**	**0.48**	**< 0.0001**
**RRCV/BSA**	**0.41**	**< 0.0001**
**mGFR of DK**	**0.29**	**0.0036**
**mGFR of RK**	**0.41**	**< 0.0001**
**Weight of DK**	**0.015**	**0.88**

*eGFR* estimate glomerular filtration rate, *CrCl* creatinine clearance, *BW* body weight, *BMI* body mass index, *BSA* body surface area, *DRV* donated renal volume, *DRCV* donated renal cortex volume, *RRV* remnant renal volume, *RRCV* remnant renal cortex volume, *mGFR* measured glomerular filtration rate

### Receiver operating characteristic analyses between each preoperative variable and estimated glomerular filtration rate a year after donor nephrectomy

**Fig. 2:**
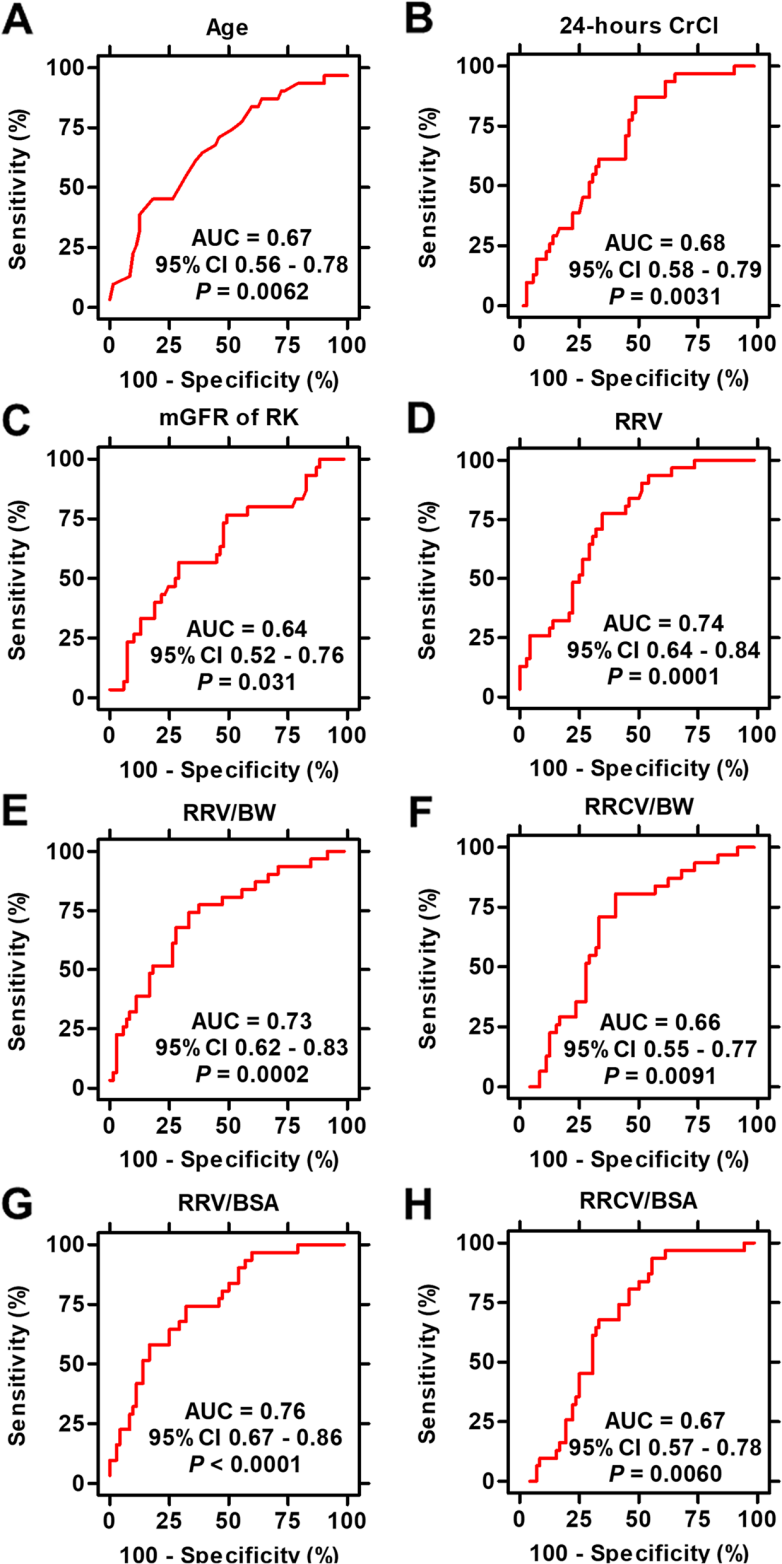
Receiver operating characteristic curve analysis for predicting remnant renal function. Various preoperative factors were evaluated to accurately predict remnant renal function (RRF). Factors involved in renal volume predicted RRF reasonably (D; remnant renal volume [RRV], E; RRV/body weight [BW], G; RRV/body surface area [BSA]), while factors involved in renal cortex volume could not predict RRF compared to factors involved in renal parenchymal volume (F; remnant renal cortex volume [RRCV]/BW, H; RRCV/BSA). The glomerular filtration rate, as measured by renal scintigraphy, could not predict RRF accurately©. CrCl: Creatinine clearance, mGFR: Measured glomerular filtration rate, RK: Remnant kidney, AUC: Area under the curve, CI: Confidence interval

ROC analyses were performed to evaluate the most effective factor for accurately predicting RRF. ROC curves, in which the value for sensitivity is plotted against the false-positive rate (1-specificity), were generated to compare the performance of each factor. The factors were evaluated to determine whether RRF was preserved to > 60% of the preoperative eGFR. Age, body composition index, preoperative renal function, split renal volume, and split renal function were analyzed. Figure 2 shows the ROC curves of age, 24-h CrCl, and mGFR of RK, RRV, RRV/BW, RRCV/BW, RRV/BSA, and RRCV/BSA. The area under the curves (AUCs) of RRV, RRV/BW, and RRV/BSA were 0.74, 0.73, and 0.76, respectively (IQR, 0.64–0.84, 0.62–0.83, and 0.67–0.86, respectively).

### Predictive model of remnant renal function

**Fig. 3:**
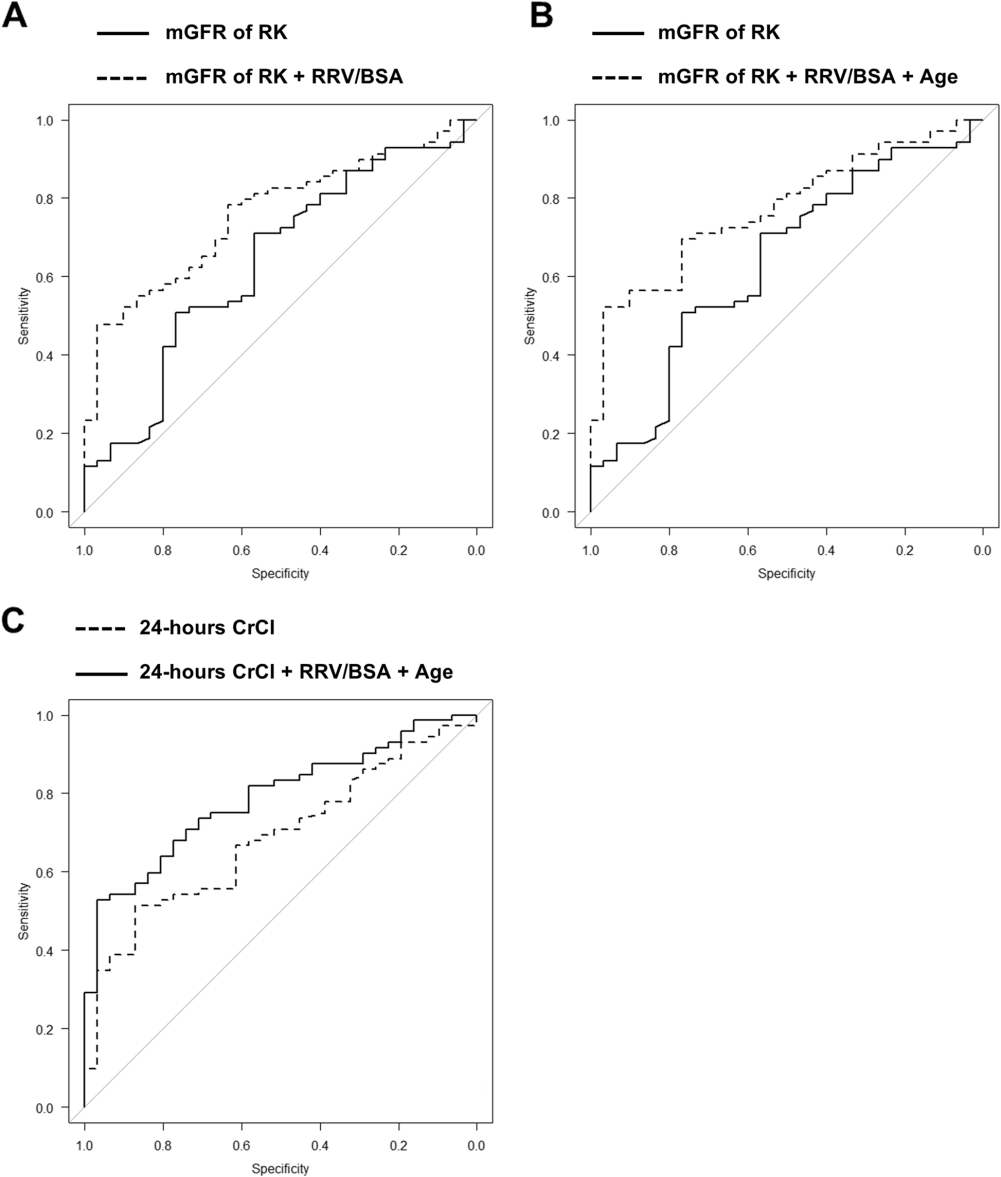
Receiver operating characteristic curve analysis to establish a predictive model for remnant renal kidney function. To establish a predictive model for remnant renal function, multivariate receiver operating characteristic curve analysis was carried out. The area under the curve (AUC) of the measured glomerular filtration rate (mGFR) of the remnant kidney (RK) was slightly low; therefore, mGFR combined with remnant renal volume (RRV)/body surface area (BSA) were analyzed, and the AUC increased compared to the AUC of mGFR (**A**: AUC, 0.76; *P* = 0.050). Furthermore, mGFR combined with RRV/BSA and age were analyzed, and the AUC significantly increased compared to the AUC of mGFR (**B**: AUC, 0.77; *P* = 0.029). In addition, 24-h creatinine clearance (CrCl) combined with RRV/BSA and age were analyzed, and the AUC significantly increased compared to the AUC of mGFR (**C**: AUC, 0.79; *P* = 0.030)

ROC analyses were carried out using EZR (Easy R), a statistical software [20], to establish a predictive model of RRF using 99 m-Tc DTPA scintigraphy findings and preoperative factors. The preoperative factors evaluated in this study were combined to increase the ROC and predict RRF more accurately. The AUC of the mGFR of the RK combined with the RRV/BSA was 0.76 (Fig. 3A) (95% confidence interval [CI], 0.66–0.86), and there was no significant difference between the mGFR of the RK and RRV/BSA (*P* = 0.050). The AUC of the mGFR of the RK combined with the RRV/BSA and age was 0.77 (Fig. 3B) (95% CI, 0.68–0.87), and there was a significant difference between the mGFR of the RK and the RRV/BSA and age (*P* = 0.029). The 24-h CrCl was analyzed with RRV/BSA and age, and the AUC was 0.79 (Fig. 3C) (95% CI, 0.70–0.88); this combination showed the highest AUC among all combinations.

## Discussion

The present study shows that volume parameters calculated by CTV and mGFR measured by 99 m-Tc DTPA scintigraphy had strong correlations with RRF. Furthermore, we also demonstrated that CTV could measure SRF and predicted RRF accurately in LKDs compared to measurement by 99 m-Tc DTPA scintigraphy. In addition, the predictive model composed of the mGFR of DK, RKV/BSA, 24-h CrCl, and age can predict RRF after DN in LKDs. Therefore, the present study suggests that CTV is a reliable assessment tool for SRF, and confirms the importance of comprehensive assessment for determining the kidney to be procured.

The highest concern is the risk of developing ESRD in LKDs after DN. A previous meta-analysis showed that 12% of LKDs developed a GFR between 30 and 59 mL/min, 0.2% of LKDs developed a GFR ≤30 mL/min, and LKDs tended to develop proteinuria more often compared to the control. Therefore, LKDs might have a slightly increased risk of developing severe CKD and ESRD compared to the healthy population [2, 3, 5, 22, 23]. Furthermore, Ibrahim et al. reported that ESRD developed in 11 LKDs at a rate of 180 cases per million persons per year compared to a rate of 268 per million per year in the general population [24]. With regards to the survival of LKDs, the majority of previous studies reported that the long-term risk of mortality was no higher for LKDs than for an age-, sex-, and comorbidity-matched general population [24–26]. Okamoto et al. reported that, in a cohort of 481 LKDs, an 84-year-old LKD died of ESRD 8 years after DN [25]. In addition, progression of CKD and proteinuria generally increases the risk of cardiovascular heart disease [27–29]. Thus, the determination of the kidney to be procured should be performed after careful consideration to avoid death due to ESRD and to decrease complications induced by CKD.

Development of comprehensive assessment tools and models for predicting SRF may make selection of the kidney to be procured safe and easy, and CTV can play an important role in evaluating the SRF. Traditionally, renal scintigraphy has been used for evaluation of SRF, and provides important information about SRF. A previous report that compared CTV and renal scintigraphy showed that SRF measured by CTV could provide equivalent information to that obtained from renal scintigraphy [6, 7]. Barbas et al. suggested that CTV was superior to renal scintigraphy in predicting RRF after DN in LKDs [17]. If CTV can evaluate SRF in LKDs as an alternative to renal scintigraphy, LKDs would be exposed to less radiation exposure. CT examination has potential value for both structural and functional information. In addition, Okumura et al. proposed a prediction model of RRF in LKDs that comprised age, sex, hypertension, and RRV/BW; the ROC analysis of which showed strong diagnostic accuracy for predicting favorable RRF [8]. The present study showed that age, RRV/BSA, and 24-h CrCl could improve the predictive accuracy of RRF compared to evaluation of 99 m-Tc DTPA alone. In our institute, SRF are assessed by both CTV and renal scintigraphy and used to determine the kidney to be procured at present, while in the near future we plan that CTV would replace renal scintigraphy, and renal scintigraphy would be used in LKDs that CTV alone is difficult to determine the kidney to be procured. Additional studies should evaluate the predictive utility of CTV, its potential role in LKD assessments, and establish a sophisticated predictive model of RRF in LKDs.

Despite advances in preoperative examination, surgical tools, and surgical techniques, minor perioperative complications occur in 10–20% of DN, major complications defined by the Clavien grading system for surgical complications level 4 or 5 occur in < 3%, and the risk of perioperative death is < 0.03% [30]. A report from the United States demonstrated that, despite chronological changes in practice and selection, surgical mortality of LKDs was 3.1 per 10,000 LKDs, and there was no change during the last decade and a half [26]. Although the Japanese guidelines for LKD have few documents about SRF and selection criteria for the kidney to be procured [31], the Organ Procurement and Transplantation Network (OPTN) Policy from the United States describes detailed medical evaluation requirements for LKDs [32]. Notably, the OPTN Policy states the considerations pertaining not only to SRF but also anatomical suitability for KT when the kidney to be procured is selected. Since CT images can provide anatomical information and lead to safe DN, CT is considered to have an essential role prior to DN.

The present study has some limitations. Despite of an inaccurate estimation for renal function eGFR was used as a measure of RRF for analysis. In the Japanese guidelines for LKD eGFR is described as criteria for renal function, therefore eGFR was chosen as a measure of RRF [31]. The data of LKDs were obtained from a single institution, and the sample size was small. In the future we have to increase the sample size and alter the weighting of individual parameters to increase accuracy predicting RRF. Furthermore, the follow-up period was short to adequately evaluate RRF and prognosis in LKDs. In this study, 99 m-Tc DTPA scintigraphy was used for renal scintigraphy, and additional analysis using a MAG3 scan is needed to provide evidence-based recommendations. Further research is warranted to establish an optimal assessment protocol for SRF.

## Conclusions

CTV can play an important role in predicting RRF and a comprehensive assessment including not only renal scintigraphy but also CTV can predict RRF accurately in LKDs before DN. Further investigation is needed to establish assessment tools and models for determining the kidney to be procured. The knowledge and understanding of the potential ability of CTV, and amount of information measured by CTV can lead to better selection criteria, resulting in preservation of RRF and improvement of survival in LKDs.

## Supplementary Information

**Additional file 1 **: **Table S1**. Clavien classification of surgical complications.

## Data Availability

The datasets generated and/or analyzed during the current study are not publicly available due to our hospital policy but are available from the corresponding author on reasonable request.

## Abbreviations

LDKT: Living donor kidney transplantation
KT: Kidney transplantation
LKD: Living kidney donor
DN: Donor nephrectomy
DK: Donated kidney
SRF: Split renal function
RRF: Remnant renal function
ESRD: End-stage renal disease
CT: Computed tomography
CKD: Chronic kidney disease
CTV: CT volumetry
DTPA: Diethylenetriamine penta-acetic acid
GFR: Glomerular filtration rate
eGFR: Estimated GFR
BSA: Body surface area
BW: Body weight
mGFR: Measured GFR
ROC: Receiver operating characteristic
IQR: Interquartile range
DRV: Donated renal volume
RRV: Remnant renal volume
DRCV: Donated renal cortex volume
RRCV: Remnant renal cortex volume
RK: Remnant kidney
CrCl: Creatinine clearance
AUC: Area under the curve
CI: Confidence interval
