# Supplementary material for: Remnant renal volume can predict prognosis of remnant renal function in kidney transplantation donors: a prospective observational study
Source: BMC Nephrol. 2021 Nov 6;22:367. doi: 10.1186/s12882-021-02568-8 (PMC8572493; doi:10.1186/s12882-021-02568-8)
Supplement: Supplementary file 1 — Additional file 1 : Table S1. Clavien classification of surgical complications. [file 12882_2021_2568_MOESM1_ESM.docx]

| **Table S1. Clavien classification of surgical complications** | |  |
| --- | --- | --- |
| **Grade** | **Definition** |  |
|  |  |  |
| **I** | **Any deviation from the normal postoperative couse without the need for pharmacological treatment  or surgical, endoscopic, and radiological interventions. Allowed therapeutic regimens are: drugs as antiemetics, antipyretics, analgetics, diuretics, electrolytes, and physiotherapy. This grade also includes wound infections opened at the bedside.** |  |
| **II** | **Requiring pharmacological treatment with drugs other than such allowed for grade I complications.** |  |
|  | **Blood transfusions and total parenteral nutrition are also included.** |  |
| **III** | **Requiring surgical, endoscopic or radiological intervention.** |  |
| **a** | **Intervention not under general anesthesia.** |  |
| **b** | **Intervention under general anesthesia.** |  |
| **IV** | **Life-threatening complication (including CNS complication) requiring IC/ICU management.** |  |
| **a** | **Single organ dysfunction (including dialysis)** |  |
| **b** | **Multiorgan dysfunction** |  |
| **V** | **Death of a patient** |  |
| **Suffix "d"** | **If the patient suffers from a complication at the time of discharge, the suffix "d" is added to the  respective grade of complication.  This label indicates the need for a follow-up to fully evaluate the complication.** |  |
| **CNS = central nervous system; IC = intermediate care; ICU = intensive care unit** | |  |
